# A Genome-Wide Transcriptional Analysis of Yeast-Hyphal Transition in *Candida tropicalis *by RNA-Seq

**DOI:** 10.1371/journal.pone.0166645

**Published:** 2016-11-16

**Authors:** Yuan Wu, Yin-hu Li, Shuan-bao Yu, Wen-ge Li, Xiao-shu Liu, Lei Zhao, Jin-xing Lu

**Affiliations:** 1 State Key Laboratory of Infectious Disease Prevention and Control, Collaborative Innovation Center for Diagnosis and Treatment of Infectious Diseases, National Institute for Communicable Disease Control and Prevention, Chinese Center for Disease Control and Prevention, Chang bai Road 155, Chang ping District, Beijing, China; 2 Microbial Research Department, BGI-Shenzhen, Main building, Beishan Industry Zone, Yantian District, Shenzhen, China; 3 Department of Molecular Physiology and Biophysics, Holden Comprehensive Cancer Center, University of Iowa Carver College of Medicine, Iowa City, IA, 52242, United States of America; Yonsei University, REPUBLIC OF KOREA

## Abstract

*Candida tropicalis* is considered as the leading pathogen in nosocomial fungemia and hepatosplenic fungal infections in patients with cancer, particularly in leukemia. The yeast-filament transition is required for virulent infection by *Candida*. Several studies have explored the genome-wide transcription profile of *Candida*, however, no report on the transcriptional profile of *C*. *tropicalis* under yeast-filament transition has been published.

In this study, the transcriptomes of three *C*. *tropicalis* isolates with different adhesion and biofilm formation abilities, identified in our previous studies, were analyzed in both the yeast and filament states using RNA-Seq. Differentially expressed genes were found for each isolate during the transition. A total of 115 genes were up- or down- regulated in the two hyphal-producing isolates (ZRCT 4 and ZRCT 45). Among these differentially expressed genes, only two were down-regulated during the yeast-filament transition. Furthermore, six filament-associated genes were up-regulated in the hyphae-producing isolates. According to *Candida* Hypha Growth Database established in this study, 331 hyphae- related genes were discovered in *C*. *tropicalis*. *ALS1* and *ALS3* were down-regulated and up-regulated, respectively, during filamentous growth of *C*. *tropicalis*. These findings proved a better understanding of gene expression dynamics during the yeast-filament transition in *C*. *tropicalis*.

## Introduction

The incidence of fungal nosocomial infections (FNIs) has increased significantly over recent decades and will likely continue to grow in importance because of the constantly increasing number of patients at risk in medical care units [1]. *Candida* species account for approximately 80% of FNIs, are considered the fourth leading cause of blood stream infections and are responsible for the overwhelming majority of urinary tract infections [2]. *Candida albicans* is the most important *Candida* species and has received major clinical attention. However, FNIs caused by non-*Candida albicans Candida* (NCAC) are increasing. *C*. *tropicalis* is frequently isolated from hospitalized patients in intensive care units (ICUs), especially in patients with cancer [3]. Furthermore, *C*. *tropicalis* is thought to be the first or the second most frequently found NCAC species in the bloodstream (candidemia) and urinary tract (candiduria) infections [4]. *C*. *tropicalis* displays the highest dissemination ability in the neutropenic host among NCAC species, even higher than that of *C*. *albicans* [5,6], which might explain the reported relatively high mortality associated with *C*. *tropicalis* infection. Consistent with *C*. *albicans*, *C*. *tropicalis* is diploid and belongs to the CUG clade, in which the CUG codon is translated as serine rather than leucine [7]. Moreover, both *C*. *albicans* and *C*. *tropicalis* can switch between a budding yeast form and an elongated, filamentous form. However, in contrast to the parasexual behavior of *C*. *albicans*, no mating was observed in *C*. *tropicalis* [8].

Adhesion, biofilm formation (BF), yeast-filament transition and secretion of hydrolytic enzymes, including secreted aspartyl proteases (Saps), esterases, lipases, phospholipases, and hemolysins, are considered as the key virulent factors of *Candida* [9]. *ALS1*-*3*, *LIP1*-*10*, and *SAP1*-*4* encode adhesins, lipases and Saps of *C*. *tropicalis*, respectively [10]. However, their specific roles in infection still remain unclear. It is reported that the gene families involved in filamentous growth of *C*. *albicans* may be different to those in *C*. *tropicalis*. In addition, *C*. *tropicalis* shows strain-dependent enzyme activity, adhesion and BF ability [11]. The yeast-filament transition plays a key role in *C*. *albicans* infection, however, its role in *C*. *tropicalis* infections of humans remains largely unknown.

The first whole genome sequence of *C*. *tropicalis* was published in 2009, and is 14.5 Mb in size, containing 6,258 genes, which represents slightly more coding genes than were assigned to the *C*. *albicans* genome [7]. Sequencing of cDNAs derived from RNA samples (RNA-Seq) provides accurate measure of the transcriptional landscape, allowing the identification of exons and introns, alternative splicing and single nucleotide polymorphisms (SNPs) during gene prediction [12]. Furthermore, cDNA sequences are helpful to optimize the annotation of some fungal genomes with limited information in GenBank, and to identify new transcripts, which is important in the study of fungal pathogenesis [13]. In addition, comparing transcriptional profiles under different conditions i.e. yeast vs. hyphae, could identify differentially expressed genes (DEGs) and further illustrate hyphal production. RNA-Seq has been applied to quantify the transcriptional landscape in many kinds of yeasts and filamentous fungi, such as *C*. *albicans* [14], *C*. *glabrata* [13], *C*. *parapsilosis* [15], *C*. *dubliniensis* [16], *Aspergillus fumigatus* [17], *A*. *niger* [18], *A*. *flavus* [19] and *A*. *oryzae* [20].

Although transcriptome studies are helpful to understand the biology of infections caused by pathogenic fungi, studies investigating the transcriptome of *C*. *tropicalis* under relevant conditions are relatively rare. To study the transcriptional landscape of the pathogenic fungus *C*. *tropicalis* comprehensively, we performed an RNA-Seq assay for three isolates with different adhesion and BF abilities, which were reported in our previous study, under yeast and hyphal conditions *in vitro* [21]. The three isolates were all isolated from sputum in different departments in one hospital. They showed moderate levels of hydrolytic enzymes and strain-dependent adhesion and BF abilities. Our study provided a list of significant DEGs common for the hyphal-producing isolates, which will offer clues for further studies in its role in *C*. *tropicalis* infection.

## Materials and Methods

### Culture and hyphal-inducing conditions

Yeast extract-peptone-dextrose (YEPD) medium was used as the standard non-filament-inducing medium, which contains 2% yeast extract, 2% peptone and 1% glucose. The hyphal form of *C*. *tropicalis* was induced in filament-inducing media, which comprised liquid YEP plus 0.75% dextrose, 50% fetal bovine serum (FBS) and synthetic defined (SD) medium (6.7 g/liter yeast nitrogen base without amino acids). Three *C*. *tropicalis* isolates, ZRCT4, 45 and 52, were firstly inoculated on Sabouraud dextrose agar media (SDA) media plate at 25°C for 24h. Following this, one clone of each isolate was picked and cultured in 10 ml YEPD media at 30°C, with shaking at 120rpm for 12 hours. To obtain the non-filamentous state, 3 ml yeast suspension was added into 30ml YEPD and then incubated at 30°C, 200rpm for 2 hours. At the same time, 1 ml of the yeast suspension was added into 30 ml of inducing media at 37°C, 200rpm for 2 hours, to obtain the filamentous cells.

### RNA isolation and RNA sequencing

Yeast cells were centrifuged at 5000 rpm, 4°C for 6 minutes in order to get the total RNA. Total RNA of these three *C*. *tropicalis* isolates in the non-filament and filament form was prepared using the RNeasy Mini kit (QIAGEN), according to the manufacturer’s protocol. DNase treatments were performed using RNase-free DNase I (QIAGEN). The purified total RNA was cut into short fragments (about 200~700 bp) using fragmentation buffer. The first-strand cDNA was synthesized using random hexamer-primers. The double strand cDNA was purified with QiaQuick PCR extraction kit (QIGEN), which were further subjected to end-polishing. Sequencing adapters were added to the ends of the fragments, which were then enriched by PCR amplification. Finally, the library products were subjected to DNA sequencing using an Illumina HiSeq 2000 appartus.

### Detection of DEGs, alternative splicing and SNPs

The filtered clean sequencing data was aligned to the genes of the reference *C*. *tropicalis* (MYA-3404) genome deposited in GenBank (http://www.ncbi.nlm.nih.gov/genome/?term=candida+tropicalis) using bowtie2 (http://bowtie-bio.sourceforge.net/bowtie2/index.shtml). The number of maximum mismatches and N bases in the reads were set at 1, and the mode of “sensitive” was chosen during the alignment. The.bam files were then used to calculate the gene expression levels using RNA-Seq by Expectation Maximization (RSEM with default parameters), and the fragments per kilobase of transcript per million mapped reads (FPKM) values of genes were obtained. TopHat was used to predict alternative splicing, with default parameters, and the expressions of different mRNA transcripts were also calculated. GATK was used to detect the SNPs in the genome, and SNPs that were supported by less than four reads were filtered out.

### Screening of DEGs

In the study, Noiseq software package [22]was selected to screen for DEGs. Firstly, the background noise was calculated for every two groups. Using the obtained FPKM values, the averaged expression value of genes in each group was obtained. The expression difference of the genes between two groups was then quantified. If the expression difference between two groups deviated from their background noise, the gene was selected as an initial DEG. Finally, a gene whose difference ratio was higher than 2 and had a deviation value higher than 0.8 was retained as a DEG between the two groups.

### Prediction of novel transcripts

Transcripts were assembled from reads which aligned to the *C*. *tropicalis* genome using Cufflink (http://cufflinks.cbcb.umd.edu/faq.html#fpkm). After comparing them with the reference genome, the assembled transcripts that were located 200bp away from any reference genes were retained. The transcripts whose lengths were over 180 bp and that were supported by more than 2 reads were defined as novel transcripts. To study the functions of the novel transcripts, the software Coding Potential Calculator (CPC http://cpc.cbi.pku.edu.cn/) was selected to assess their protein-coding ability.

### Hypha-related genes in *C*. *tropicalis*

A *Candida* Hyphal Growth Database was established using hyphal-related genes obtained from Candida Genome Database (www.candidagenome.org), including *C*. *albicans*_SC5314A22, *C*. *dubliniensis*_CD36, *C*. *glabrata* CBS138 and *C*. *parapsilosis*_CDC317. The reference genes and novel transcripts of *C*. *tropicalis* were then aligned to the database using blastp (identity > 40% and e-value < 1e-5) and the genes related with hyphal growth were obtained.

### Quantitative real-time reverse transcription PCR (qRT-PCR)

To evaluate the validity of the RNA-Seq data, three gene families *ALS* (*ALS1*, *2*, *3*), *LIP* (*LIP1*, *4*), and *SAP* (*SAP1*, *2*, *3*, *4*) were tested in isolates ZRCT52 and 45 under yeast and hyphal status, respectively, using RT-PCR. RNA extraction was performed as in “RNA isolation and RNA sequencing” above. The purified RNA was converted to cDNA using a Go Script^TM^ Reverse Transcription System kit (Promega). The primers used for these genes were the same as those used in a previous study [23]. *ACT1* was used as an internal control. The reaction system comprised 10 μl Power SYBR^®^ Green PCR Master Mix (Applied Biosystems), 1 μM forward and reverse primer and 1 μl of cDNA, in a final reaction volume of 20 μl. Negative (commercial RNA and DNA free water) were included in each run. All the RT-PCR reactions were performed on the 7500 Fast Real-Time PCR System (Applied Biosystems). Fold changes in gene expression were determined using the ΔC_T_ method and data were normalized against the expression of *ACT1* [23]. Each reaction was performed in triplicate and mean values of the relative expression were analyzed for each gene.

## Results

### Summary of sequencing data and mapping of the *C*. *tropicalis* genome

**Fig 1:**
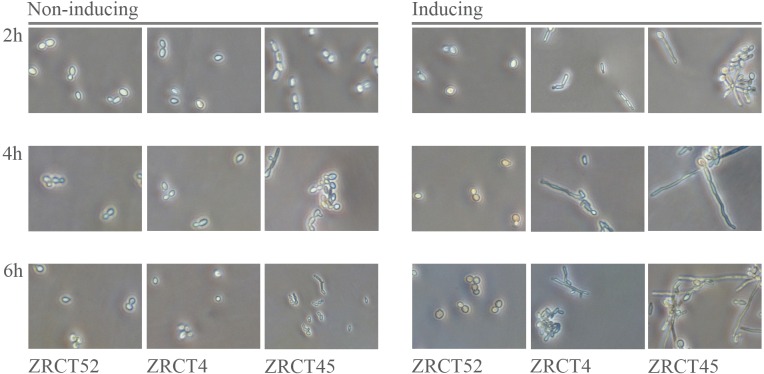
Yeast-hyphal status of three *C*. *tropicalis* isolates under inducing condition under microscopy. Each isolate was cultured and induced at three time points (2h, 4h, 6h).

To obtain complete transcriptome data, which is a representative of the *C*. *tropicalis* genome, we prepared mRNAs from three *C*. *tropicalis* isolates ZRCT52, 4 and 45 with different hyphae inducing abilities (Fig 1). The result displayed that ZRCT52 does not form hyphae, while ZRCT4 shows intermediate hyphal formation and ZRCT 45 is hyper filamentous. Each isolate was tested under both yeast and hyphal-inducing conditions. The total reads covered at least 88% of the reference genome of *C*. *tropicalis* in all six analyzed samples. (Table 1). The number of transcripts detected was slightly higher under hyphal-inducing conditions for all isolates. Detailed information is shown in Table 1. Around 53% of these *C*. *tropicalis* sequence reads were mapped successfully to the reference genomic sequences at corresponding positions, with perfect matches or 1–2 mismatches using RMAP (http://rulai.cshl.edu/rmap/) (for details see the materials and methods section). In these six samples, the transcriptional profile of hyphae was obviously different from that of yeast cells, with a substantial number of genes being up-regulated. Moreover, these changes were different among the three isolates, including genes involved in the transition, exons, SNP, novel transcripts and alternative splicing. Firstly, DEGs between the two different forms (yeast and hyphae) within each strain were analyzed. In a gene ontology (Go) functional classification analysis, most DEGs in the ZRCT4 and ZRCT 52 yeast-hyphal groups were gathered in “Cellular process”, “Metabolic process”, and “catalytic activity”, while the altered genes in the ZRCT 45 yeast-hyphal group were in “Cellular process”, “Metabolic process”, and “Binding” categories. There were more DEGs in the 45 yeast-hyphal group than in the other two groups. Statistics of the top 20 pathways enriched for the 3 groups were studied, respectively. Target genes in the ZRCT 45 yeast-hyphal group showed significantly different pathways from ZRCT 52 yeast-hyphal and ZRCT 4 yeast-hyphal groups. Almost 30 genes of ZRCT 45 were clustered in the purine metabolism pathways. Genes enriched in ZRCT 4 and 52 were relatively dispersed. Three notable pathways were found in ZRCT 4: “glycine serine and threonine metabolism”, “arginine and proline metabolism”, and “alanine aspartate and glutamate metabolism”. In addition, the pathways of alcoholism and systemic lupus erythematosus were obviously enriched among the target genes of the ZRCT 52 group. This may indicate that distinct pathways are involved in the hyper-filamentous strain and non-hyphae forming strain.

**Table 1:** Summary of 6 transcriptional profiles.

Sample Name	Clean reads	Genome map Rate %	Gene map Rate %	ExpressedGene	Expressed Transcripts	Expressed Exon	Extend Gene	90–100% mapped exons
Ctro4-hypha	33651792	89.09	53.34	5873	5873	9438	1688	9385
Ctro4-yeast	36328486	88.38	52.35	5746	5746	8750	1375	10435
Ctro45-hypha	36481428	88.24	58.53	5887	5887	9958	1833	7368
Ctro45-yeast	36887808	88.06	53.91	5810	5810	9256	1528	8009
Ctro52-hypha	36658176	89.99	61.86	6002	6002	11623	1635	7890
Ctro52-yeast	36366766	88.25	55.89	5992	5992	10703	1800	8641

**Fig 2:**
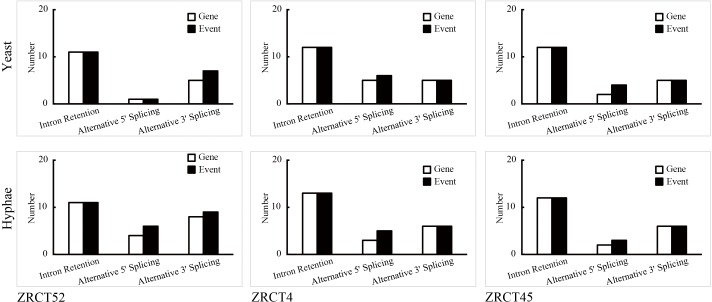
The alternative splicing of three isolates (ZRCT 52, ZRCT4, ZRCT 45) under yeast and hyphae status. “Gene” refers to number of genes involved in alternative splicing. “Event” means frequency of types of splicing found in one gene, such as intron retention, alternative 5’ splicing and alternative 3’ splicing.

**Fig 3:**
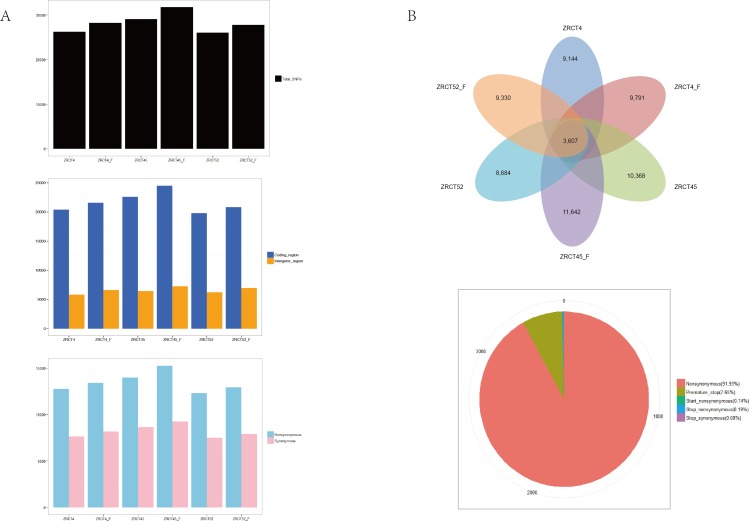
The SNP analysis of three isolates of yeast and hyphae at transcriptional level, respectively. (A) Total SNP, number SNP located in coding region and intergenic region, non-synonymous/synonymous in three isolates of both yeast and hyphae. (B) Common SNP in three isolates of both yeast and hyphae. The pie graph displayed the types of SNP found in this study.

We identified 8750–11623 expressed exons in the six transcriptional files. Exons that covered the reference genome of MYA-3404 with 90–100% identity represented more than 80% of all identified exons (Table 2). Furthermore, alternative splicing, including exon skipping, intron retention, alternative 5’-splicing and alternative 3’-splicing were analyzed for expressed transcripts in the six samples (Fig 2). The numbers of genes involved in alternative splicing events were similar in all the six transcriptional profiles. No exon skipping was found in the six samples. More intron retention was observed compared with the other two types of alternative splicing (Fig 2). However, except in ZRCT 52 yeast, more than one splicing events was found in one identified gene with alternative splicing for alternative 5`-splicing of all sequenced samples. In addition, more than one splicing events was found per gene for alternative 3`-splicing only in the ZRCT 52 yeast and hyphal transcriptional samples. Among the six samples, we only detected one example of intron retention in one gene (Fig 2). More than one hundred novel transcripts were discovered in the analyzed samples, most of which were located in non-coding regions (Table 2). Tens of thousands of SNPs were found spread over the whole genome, most of which were located in coding sequence (CDS) regions (Fig 3A). For all transcripts, more SNPs were identified in hyphae than in yeast cells. The sequential order is of high to low number of SNPs was ZRCT45, ZRCT 4, and ZRCT 52 (Fig 3A), which correlated with the order of hyphae forming ability *in vitro*. The majority of SNPs were non-synonymous among all six samples (Fig 3A). The ZRCT45 hyphal sample had the most SNPs, including non-synonymous and synonymous SNPs. There were 3,607 shared non-synonymous SNP among the six transcriptional profiles, comprising 276 premature stop codons, 5 start non-synonymous, 7 stop nonsynonymous, 3 stop synonymous, and 3,316 non-synonymous SNPs (Fig 3B). Thirty-one out of 3,607 shared non-synonymous SNPs were found in DEGs from three groups, ZRCT52 yeast-hyphal, ZRCT4 yeast-hyphal, and ZRCT45 yeast-hyphal (Fig 3). Based on the comparative analysis, we found that the three isolates showed specific transcriptional profiles under yeast and hyphal conditions, which might provide an explanation of their differences in adhesion, BF and hyphal-inducing ability. Moreover, different SNPs found in these samples might be used as specific site to study the genetic features of *C*. *tropicalis* populations, and will help in further explore the mechanism of yeast-hyphal transition.

**Table 2:** Main characters of six transcriptional profiles.

Sample	No. of novel transcript	No. of novel coding genes	No. of total SNP	No. of SNP in intergenic region	Alternative Splicing
52yeast	142	22	25884	6241	24
52hyphal	132	22	27596	6970	23
4yeast	144	24	28065	6629	21
4hyphal	113	23	26106	5832	21
45yeast	133	20	28886	6449	26
45hypal	123	29	31594	7272	19

### Comparative analysis of three *C*. *tropicalis* isolates in yeast and hyphal status at the transcript level

**Fig 4:**
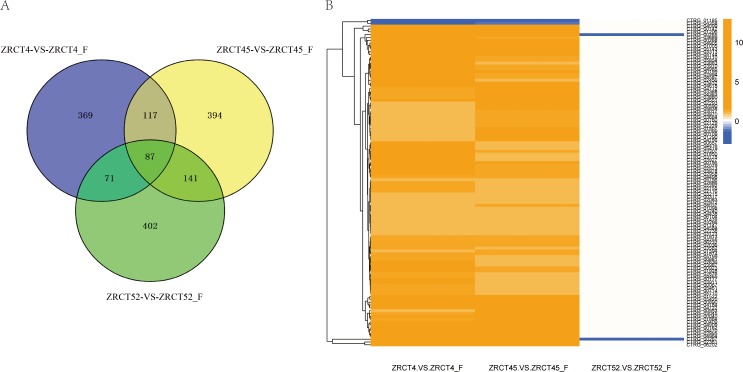
Gene expression of *C*. *tropicalis* during yeast-hyphae transition of three isolates. (A) Number of differentially expressed genes in hyphae inducing condition of *C*. *tropicalis* among three groups, ZRCT4, ZRCT45, and ZRCT 52 yeast-filamentation groups. (B) Cluster diagram of common genes involved in yeast-hyphae transition of ZRCT4 and ZRCT45. Blue, reduced expression; yellow, increased expression.

**Fig 5:**
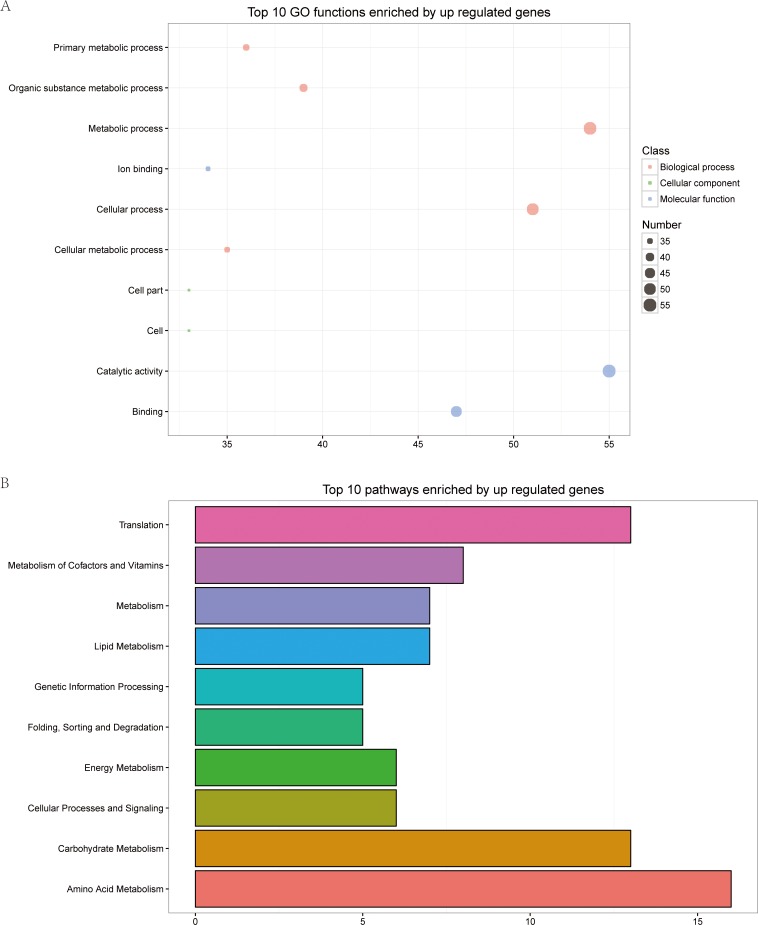
Pathway enrichment analysis of DEGS and GO functional classification of common genes within three yeast-hyphae transition groups. (A) Top 10 GO functions enriched by up-regulated genes, respectively. (B) Top 10 pathways enriched by up-regulated genes, respectively.

To explore the possible mechanism of the yeast-hyphal transition, we compared the transcriptional profiles between the yeast and hyphal forms of three isolates, ZRCT52, ZRCT4, and ZRCT45. A total of 701, 644 and 739 genes were differentially expressed during yeast-hyphal transition in ZRCT52, ZRCT4 and ZRCT45, respectively. There were more up-regulated genes than that down-regulated genes in all three isolates, with 131, 37 and 120 down-regulated and 570, 607 and 619 up-regulated genes in ZRCT52, ZRCT4 and ZRCT45, respectively (Fig 4A). ZRCT45, with the strongest hyphal-inducing ability *in vitro*, had the largest number of DEGs. Interestingly, ZRCT52 produced almost no hyphae in the inducing experiment; however, DEGs were still observed between the two conditions (Fig 4A). We further analyzed the common DEGs among the 4 and 45 yeast-hyphal groups. As a result, 115 commonly expressed genes were identified (Fig 4B), within which two transcripts (CTRG_01185 and 04358) were down-regulated, and the other 113 genes were up-regulated (S1 File). Among the 115 genes, most were detected as un-changed in ZRCT 52, and there were only two genes (CTRG_00267 and 00268) that were down-regulated in the ZRCT 52 yeast-hyphal transition (Fig 4B and S1 File). These common 115 genes don’t include DEGs common to all the three strains. This suggested that these 115 genes play important roles in the yeast-hyphae transition of *C*. *tropicalis*, because isolate ZRCT 4 and ZRCT 45 are hyphal-producing, while ZRCT52 produce no hyphae. Eighty-four genes showed the same changing trend between the three isolates within yeast and hyphae conditions, while three genes (CTRG_00265, 00267 and 02858) showed different expression patterns in these three groups. Furthermore, the expressions of CTRG_00265 and 00267 were down-regulated in the yeast-hyphal transition of ZRCT52, but were up-regulated in the transition of ZRCT 4 and 45. When searched in GenBank, CTRG_00265 and 00267 are partial sequences located within an exon, which is similar to repressed by TUP1 protein, while CTRG_02858 is similar to potential mitochondrial protein Fmp27. TUP1 was recognized as an important transcriptional repressor of *C*. *albicans*, deletion of which would cause the cells to be locked in the yeast cell form. This feature may partially explain the lack of hyphae forming ability of ZRCT52.By contrast, ZRCT 4 and 45 could form moderate or high numbers of hyphae, respectively. The top ten pathways enriched by common up-regulated genes within the ZRCT 4 and 45 yeast-hyphal groups are shown in Fig 5B. Fifteen up-regulated genes were significantly enriched in the amino acid metabolism pathways. The gene number are as follows: CTRG_04587, 03178, 05710, 05419, 05854, 03286, 01594, 03911, 02116, 00760, 01005, 03045, 03432 and 01206. Moreover, the only two down-regulated genes (CTRG_01185 and 04358) were involved in MAPK and metabolic pathways, respectively. The GO functions analysis, revealed that more than 45 up-regulated genes were obviously enriched in the metabolic process, cellular process, catalytic activity and binding categories (Fig 5A).The down-regulated genes were enriched in the cellular component category.

In *C*. *albicans*, *ALS3* and *SAP 4*–*6* were reported to be involved in hyphal development [24]. *SAPs* were highly expressed after 6-h of culture in serum-containing medium at 37°C, but were only mildly induced at the 30- and 60-min time points [25]. The *ALS3* gene of *C*. *albicans* was highly expressed in strains with high biofilm formation ability (HBF) and during hyphae-inducing conditions [26]. Different ALS proteins promote *C*. *albicans* isolates to adhere to different types of epithelial cells [24]. Here, we explored the transcription levels of *ALS1*-*3*, *LIP1*, *4*, and *SAP1*-*4* of *C*. *tropicalis* isolate ZRCT52, 4 and 45 in the yeast and hyphal states. *ALS1* was down-regulated in isolate 4 and 52 during the yeast-hyphal transition, but not in isolate 45. *ALS2* showed no changes in these three strains, while *ALS3* was up-regulated in ZRCT 45 and ZRCT4. Our results suggested that *ALS3* plays an import role in the filamentation of *C*. *tropicalis*, which is consistent with its role in *C*. *albicans*. *LIP1* was down-regulated in ZRCT52, but up-regulated in ZRCT45. Furthermore, *LIP4* was up-regulated in both ZRCT52 and ZRCT45. Except for *SAP1*, all members of the *SAP* gene family were up-regulated in the three strains. This suggested *SAP*2 and *SAP3* are important during the yeast-hyphal transition of *C*. *tropicalis* under inducing condition *in vitro*. The gene expressions of *ALS1*-*3*, *LIP1*, *4*, and *SAP1*-*4* of *C*. *tropicalis* under yeast and hyphae-inducing condition were detected by qRT-PCR. Except for *ALS2*, the tested genes displayed the same change trend in the RNA-seq and qRT-PCR analyses. The expression *ALS2* was unchanged in the RNA-seq analysis, but was detected as down-regulated in ZRCT52 and 4, and up-regulated in ZRCT45 by qRT-PCR.

### Analysis of hyphae-related genes in *C*. *tropicalis*

According to the *Candida* Hyphal Growth Database constructed in this study, which contains 904 hyphae-related genes, 331 hyphae-related genes were identified in *C*. *tropicalis* isolate, ZRCT4 (S2 File). Among these genes, twenty-three were up-regulated, and might play an extremely important role in hyphal growth, filamentation and invasive growth of *C*. *tropicalis*, and the remainder were unchanged (S2 File). In *C*. *albicans*, some of these 23 genes were only up-regulated significantly under special inducing conditions, such as GlcNAc, antibiotics, stress, pH and NO [27]. However, in our study, they all could be induced by serum and 37°C in *C*. *tropicalis*, indicating that specific pathways are employed by *C*. *tropicalis*.

Multiple signaling and regulatory pathways control the yeast-hyphal transition in *C*. *albicans*. Based on a comparison between *C*. *albicans* and *C*. *tropicalis*, several up-regulated filamentation-associated genes of *C*. *tropicalis* were mapped onto pathways known to control morphological transition, such as *ECM4*, *CCC1*, *DUR1*, *2* and C5_02380W_A, which are regulated by *Nrg1*/*Tup1*, *Sef1*/*Sfu1*/*Hap43*, *Nrg1*/*Hap43*, and *Ssn6p*, respectively. In *C*. *albicans*, *CST 20* and *CEK1* encode components of the mitogen-activated protein kinase (MAPK) signaling pathway [28]; however, only *CEK1* has been identified in *C*. *tropicalis*. Furthermore, both *CDC 42* and *CDC24*, which function upstream of the MAPK pathway, are present in both *C*. *albicans* and *C*. *tropicalis* [29]. *HOG1*, a MAP kinase involved in several metabolic processes of *C*. *albicans*, was expressed during the yeast-hyphal transition of hyphae producing *C*. *tropicalis* isolates. In addition, *MKC1*, a downstream target of the protein kinase C (PKC) pathway of *C*. *albicans* [29], was expressed during filamentation in *C*. *tropicalis*. *PHR1*, a gene involved in the pH response signaling pathway of *C*. *albicans*, was also identified to be expressed in *C*. *tropicalis* [28]. The product of *PHR1*, Phr1, is a putative cell surface glycosidase with involvement in cross-linking of the beta-1, 3-and beta-1, 6-glucans [28].

Several genes, such as *RHB1*, *GAP1*, *MEP2* and *SIT4*, which are targets of the rapamycin (TOR) signaling pathway regulating a wide variety of cellular process under nutrient conditions [30], were also expressed in the 2-hour-hyphal-inducing process of *C*. *tropicalis*. The expressions of these hyphae-related genes are under the control of a series of transcription factors. In a previous study, 38 regulators of filamentous growth were identified by screening an overexpression library of 156 transcription factors of *C*. *tropicalis* [31], most of which demonstrated a conserved role in the regulation of filamentation, similar to their homolog in *C*. *albicans* or *Saccharomyces cerevisiae*. Moreover, a set of regulators was found to play specific roles in *C*. *tropicalis*. In our study, only one conserved regulator, *TEC1*, a transcription factor of TEA/ATTS, which promotes filamentous growth and BF in both *C*. *albicans* and *C*. *tropicalis*, was identified.

**Fig 6:**
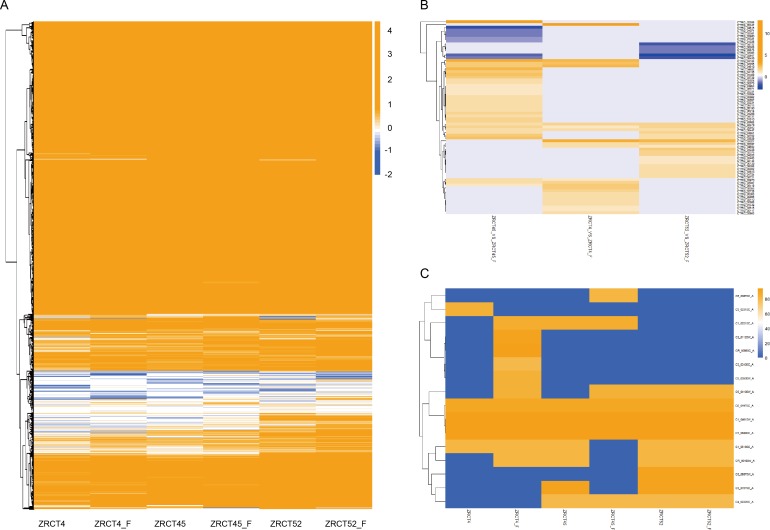
Cluster diagram of hyphae related genes in tested samples. (A) Cluster diagram of differentially expressed hyphae-588 related genes in three isolates under both yeast and hyphae status. According to *Candida* Hypha Growth Database established in this study, 331 hyphal related genes were discovered of *C*. *tropicalis*. (B) Cluster diagram of common hyphae related genes with differently expressed transcriptional level among ZRCT4, ZRCT45, and ZRCT 52 yeast-filamentation groups. A total of 70 genes were found in these three groups. (C) Cluster diagram of new transcripts related with filamentation growth in three isolates with yeast and hyphae status. Sixteen new transcripts related with filamentation growth were found here

There were 50 significantly expressed hyphae-related genes of *C*. *albicans* identified to be homologous to hyphae-related genes of *C*. *tropicalis* (S2 File). Fig 6A shows the expression profiles of these hyphae-related genes in the three *C*. *tropicalis* isolates in both yeast and hyphal forms. Differentially expressed genes in yeast-hyphal transition among these isolates were clustered in certain gene groups. However, some single genes displayed different expression trends in each isolate. A list of significantly distinctly expressed hyphae-related genes, including 70 genes among groups of ZRCT52 yeast *vs* hyphal, ZRCT4 yeast *vs* hyphal, ZRCT45yeast *vs* hyphal is shown in Fig 6B. Detailed information for these 70 genes is presented in S2 File. Novel transcripts among these 6 samples are listed in Table 2 and sixteen hyphal-related genes within those novel transcripts are shown in Fig 6C. A heat map built based on these 16 genes showed their different expressions in the 6 samples (Fig 6C). Interestingly, none of these 16 genes showed changes during yeast-hyphal transition of isolate ZRCT52.

## Discussion

The yeast-hyphal transition plays an important role in the invasive infection of dimorphic *Candida* species. Many studies have focused on the genes and regulatory pathways involved in filamentation of *C*. *albicans* [32]. In *C*. *albicans*, although the ability to produce yeast cells is critical for dissemination, it is the hyphal form that aids the species’nt survival and escape from macrophages and allows it to penetrate and invade tissue [24]. In addition, survival in serum is essential for pathogenic *Candida* species to disseminate and proliferate in host. However, limited studies have been reported about the mechanism for invasion and the role of the yeast-hyphal transition in *C*. *tropicalis* infection. In the present study, we firstly carried out a genome-wide transcriptional analysis of three *C*. *tropicalis* isolates which have different adhesion, BF, and hyphal growth abilities under the inducing conditions with serum at 37°C. For each isolate, the transcriptional profile of hyphae formed after culturing under inducing conditions for 2h was compared with that of yeast cells. Because in *C*. *albicans*, no obvious hyphae were formed at 30 and 60 minute of culturing with serum at 37°C [25]. In this study, isolate ZRCT52 couldn’t produce any pseudohyphal or hyphal no matter the inducing periods of 2h, 4h or 6h (Fig 1). Filamentation growth was increased in isolated ZRCT4 as the inducing time was prolonged (Fig 1). In isolate ZRCT45, some cells showed elongation even at YEPD culture condition for 2h, and hyphae were formed after continually cultured for 4h and 6h in YEPD (Fig 1). ZRCT45 displayed high level BF on both polystyrene (PMP) and TCC-SUP surface, and strong filamentation ability in vitro [21]. ZRCT 4 stayed at the medium level on these aspects [21]. ZRCT52 having low BF ability on PMP and medium BF on TCC-SUP, but showed no hyphal growth in vitro [21]. In our previous study, all these three isolates showed moderate activity on secreted aspartyl proteases and hemolysins [21,33]. It is clear that *C*. *tropicalis* present strain-dependent enzymatic activities. BF was apparently associated with hyphal producing ability. However, transcriptional analysis of genes implicated in BF showed no significant differential expression profiles between low BF and high BF for most related genes of *C*. *albicans* [26]. Corresponding to strain-specific morphological character of *C*. *tropicalis*, isolates ZRCT45, 4, and 52 exhibited their unique transcriptional profiles in both yeast and hyphal form (Fig 6). Furthermore, most genes related to yeast-hyphal transition are distinct to these three isolates (Fig 6). Clustering the expression of genes involved in yeast-hyphal transition of these three groups in a heat map showed their relationship with one another and variable expression within isolates (Fig 6). A set of 115 common genes of hyphal-producing isolates (ZRCT 4 and ZRCT 45) kept the same up- and down-regulated status, but showed unchanged or reverse expression in isolate ZRCT 52, which producing no hyphal. These genes were involved in variety of function and pathways. Among the common genes, only 2 genes were down-regulated and the rest genes were up-regulated. Among the 113 up-regulated genes, several genes *FET34*, *DHH1*, *ADE5*,*7*, *ENO1*, *GAL10* and *SHM2*, were identified with filamentation of *C*. *tropicalis*. These genes encoding proteins involved in several metabolic process of *C*. *albicans*, *S*. *cerevisiae* and *C*. *orthopsilosis*. In future study, we will further explore the roles of these genes in BF, filamentation, and invasive infection in animal models.

Only three *Candida* species (*C*. *albicans*, *C*. *dubliniensis*, and *C*. *tropicalis*) are capable of forming true hyphae [29]. *C*. *albicans* can undergo a morphological transition from yeast form to pseudohyphal and hyphal filaments in reaction to a wide variety of environmental conditions [29]. Pathogenic *Candida* species showed a significant expansion of gene families associated with virulence related processes (Als-like adhesion and secreted lipase genes) compared to their nonpathogenic relatives by whole-genome sequencing [7]. Approximately 105 genes were involved in filamentation of *C*. *albicans*, among which 75 genes were found to be conserved in *C*. *tropicalis* [27]. It was reported that expression of numerous genes is altered during hyphal morphogenesis. Genes *SAP4*, *5* and *6* which encode SAP proteases are well-studied examples of hypha-co-regulated virulence genes. SAP proteases have been reported to degrade host proteins, such as components of the extracellular matrix and those that are involved in host defense, which enhance the pathogenicity of *C*. *albicans* [24]. Genes *SAP 2*–*4* were up-regulated in yeast-hyhpal transition of these three *C*. *tropicalis* isolates. However, no obvious changes of *SAP1* was found. Als3p, identified as a hypha-specific cell wall protein, was not necessarily related with hyphal formation but is important in pathogenicity of *C*. *albicans* by enabling it to move out of the bloodstream, colonize in tissues, and form biofilms [30]. The *ALS* gene family, which comprised of eight members of *C*. *albicans*, and three members of *C*. *tropicalis*, encodes glycosylphosphtidylinositol (GPI)-modified cell wall glycoproteins [24]. In this study, we analyzed the expression profile of *ALS* 1–3 during the filamentation process in three *C*. *tropicalis* isolates. The expression of *ALS* 3 was up-regulated in ZRCT 4 and ZRCT45, which produced hyphae after induction at 2h point. However, no significant change in *ALS3* expression was detected of isolate ZRCT 52 with no filamentation growth after induction for 2 hours. These results indicated that *ALS* 3 might play a crucial role in hyphal growth of *C*. *tropicalis*. But how it enable *C*. *tropicalis* to realize infection needs to be further explored. Approximately 85% of the genes originally identified in the *C*. *albicans* filamentous-growth transcriptional program have homologs in non-*albicans Candida* species [29]. Compared with *S*. *cerevisiae*, *Candida* species, especially *C*. *albicans* and *C*. *tropicalis*, have a significantly expanded number of gene families that are associated with virulence, including those encode cell wall components, secreted proteins, and transporters involved in a variety of processes, such as nutrient acquisition, and energy production [7].

During the yeast-hyphal transition, expression of genes involved in a wide variety of virulence processes is coordinately controlled by a common set of signal transduction pathways and the downstream transcriptional regulators. In *C*. *albicans*, hyphal growth was controlled by several pathways: mitogen-activated protein (MAP) kinase, cAMP-protein kinase A (PKA), GlcNAc, and pH- and amino acid-sensing pathways [34]. In a recently published study, approximately 40 transcription factors regulating filamentous growth were identified in *C*. *tropicalis* under a series of inducing condition [31]. Transcription regulator, *Tec1*, was also found in our study, which demonstrate a conserved role of *Tec1* in regulation of filamentation, in consistent with their homologues in *C*. *albicans* or *S*. *cerevisiae*.

Filamentious growth play an extremely important role in pathogenicity of *Candida*. Hyphal-related genes had been widely studied in *C*. *albicans*. According to the *Candida* Genome Database (http://www.candidagenome.org), the *Candida* Hypha Growth Database was built in this study, which contains 904 hyphal-related genes. Through comparative analysis, 331 hyphal-related genes were finally identified in *C*. *tropicalis* isolate ZRCT4 (S2 File). Twenty-three genes out of them were up-regulated, while the rest genes had no change in expression. Six filamentation growth-related genes, were identified to be up-regulated in ZRCT4 and 45 yeast-hyphal transition.

In summary, comparative studies were performed in transcriptional profile of three *C*. *tropicalis* isolates with different BF and hyphal-inducing abilities in order to find core genes related with yeast-hyphal transition. The largely distinct transcriptional responses were observed between the two filament-producing strains (ZRCT4 and 45), which supplement shortness of studying response in a single genetic background (such as in yeast or hyphae form). A total of 115 common genes within ZRCT4 and 45 yeast-hyphal groups were identified, of which 6 up-regulated genes were found involved in hyphal growth of *C*. *tropicalis*. These 115 genes stayed unchanged or reversed in ZRCT 52, illustrating that they are likely to be hyphal responsive genes. According to *Candida* Hypha Growth Database built in this study, 331 hyphal-related genes were finally identified in *C*. *tropicalis* isolate ZRCT4. In future studies, we will further explore the functions of these genes in pathogenicity of *C*. *tropicalis*. The raw transcriptional data were summarized in S3 File.

## Supporting Information

S1 File Common differiated expressed genes of ZRCT4 and 45 yeast-hyphal groups. The genes in the file were having the same regulated trend in ZRCT 4 and 45, but have no changes or reverse trend in ZRCT52.
(XLSX)

S2 File Hyphal-related genes identified in *C*. *tropicalis*. According to the *Candida* Hypha Growth Database build in this study, which contains 904 hyphal-related genes, 331 genes were totally identified in *C*. *tropicalis* isolate, ZRCT4. The genes displayed differentially expressed trend in ZRCT 52 were highlighted, and those genes might be excluded in further study.
(XLSX)

S3 File The raw expression analysis data of identified expressed transcripts of 6 samples in this study. (XLSX)
